# Efficient algorithms for simulating sequences along a phylogenetic tree

**DOI:** 10.1093/bioinformatics/btaf686

**Published:** 2025-12-29

**Authors:** Elya Wygoda, Asher Moshe, Nimrod Serok, Edo Dotan, Noa Ecker, Naiel Jabareen, Omer Israeli, Itsik Pe’er, Tal Pupko

**Affiliations:** The Shmunis School of Biomedicine and Cancer Research, George S. Wise Faculty of Life Sciences, Tel Aviv University, Tel Aviv 69978, Israel; The Shmunis School of Biomedicine and Cancer Research, George S. Wise Faculty of Life Sciences, Tel Aviv University, Tel Aviv 69978, Israel; The Shmunis School of Biomedicine and Cancer Research, George S. Wise Faculty of Life Sciences, Tel Aviv University, Tel Aviv 69978, Israel; The Shmunis School of Biomedicine and Cancer Research, George S. Wise Faculty of Life Sciences, Tel Aviv University, Tel Aviv 69978, Israel; The Henry and Marilyn Taub Faculty of Computer Science, Technion—Israel Institute of Technology, Haifa 3200003, Israel; The Shmunis School of Biomedicine and Cancer Research, George S. Wise Faculty of Life Sciences, Tel Aviv University, Tel Aviv 69978, Israel; The Shmunis School of Biomedicine and Cancer Research, George S. Wise Faculty of Life Sciences, Tel Aviv University, Tel Aviv 69978, Israel; The Shmunis School of Biomedicine and Cancer Research, George S. Wise Faculty of Life Sciences, Tel Aviv University, Tel Aviv 69978, Israel; Department of Computer Science, Columbia University, New York, NY, 10027-7003, United States; The Shmunis School of Biomedicine and Cancer Research, George S. Wise Faculty of Life Sciences, Tel Aviv University, Tel Aviv 69978, Israel

## Abstract

**Motivation:**

Sequence simulations along phylogenetic trees play an important role in numerous molecular evolution studies such as benchmarking algorithms for ancestral sequence reconstruction, multiple sequence alignment, and phylogeny inference. They are also used in phylogenetic model-selection tasks, including the inference of selective forces. Recently, Approximate Bayesian Computation (ABC)-based approaches have been developed for inferring parameters of complex evolutionary models, which rely on massive generation of simulated data. For all these applications, computationally efficient sequence simulators are essential.

**Results:**

In this study, we investigate fast algorithms for simulating sequences along a phylogenetic tree, focusing on accelerating the speed-limiting component of the simulation process: handling insertion and deletion (indel) events. We demonstrate that data structures which efficiently store indel events along a tree can substantially accelerate the simulation process compared to a naive approach. To illustrate the utility of this efficient simulator, we integrated it into an ABC-based algorithm for inferring indel model parameters and applied it to study indel dynamics within *Chiroptera*.

**Availability and implementation:**

The source code for the different simulation algorithms, alongside the data used, is available at: https://github.com/nimrodSerokTAU/evo-sim. The simulator has also been integrated into SpartaABC, a website for the inference of indel parameters, accessible at: https://spartaabc.tau.ac.il/.

## 1 Introduction

The reconstruction of multiple sequence alignments (MSAs) and phylogenetic trees is at the heart of molecular evolution and genomics research. Many tools exist for both MSA (Katoh *et al.* 2002, Thompson *et al.* 2002, Löytynoja 2014) and phylogenetic tree inference (Guindon *et al.* 2009, Nguyen *et al.* 2015, Kozlov *et al.* 2019), each employing different strategies. These tools are extensively used by the scientific community, with alignment and tree reconstruction methods ranking among the most cited works in scientific literature.

To compare these tools and identify areas for improvement, benchmark datasets are needed—specifically, cases where the true MSAs and trees are known. Unfortunately, the true evolutionary history of empirical datasets is almost always unknown. Consequently, researchers commonly rely on simulated data, for which the true results are available, to compare performance (Garland *et al.* 1993, Kuhner and Felsenstein 1994, Tateno *et al.* 1994, Huelsenbeck 1995, Katoh *et al.* 2002, Fletcher and Yang 2010, Jordan and Goldman 2012, Boussau *et al.* 2013, Pervez *et al.* 2014, Kalaghatgi *et al.* 2016, Vialle *et al.* 2018, Emms and Kelly 2019).

Simulations are also integral to parametric bootstrap approaches. These approaches have been previously used in evolutionary studies to detect deviations of data from proposed models and to identify unaccounted data characteristics that may cause such deviations (Goldman 1993, Thorne *et al.* 1996). For example, Wollenberg and Atchley (2000) employed parametric bootstrap to investigate how structural and functional constraints lead to non-independent evolution of sites within a sequence.

Furthermore, simulations play a central role in Approximate Bayesian Computation (ABC) methods. ABC is an approach for inferring parameters of probabilistic models that bypasses the need for explicit likelihood calculations (Beaumont *et al.* 2002, Przeworski 2003, Tallmon *et al.* 2008, Csilléry *et al.* 2010, Templeton 2010, Kuhlwilm *et al.* 2019). This methodology relies on generating numerous simulated datasets based on models with parameters sampled from prior distributions. The accuracy of inference strongly depends on the number of simulations that can be generated (Götte 2019). We previously developed SpartaABC, an ABC-based methodology for inferring insertion and deletion (indel) evolutionary dynamics (Karin *et al.* 2017, Loewenthal *et al.* 2021, Wygoda *et al.* 2024). These studies directly motivated our current effort to develop efficient sequence simulation algorithms.

Machine learning algorithms have recently been introduced to the field of phylogenetics (Abadi *et al.* 2020, Leuchtenberger *et al.* 2020, Suvorov *et al.* 2020, Zou *et al.* 2020, Kumar and Sharma 2021, Dotan et al. 2023, Arasti and Mirarab 2024, Mo *et al.* 2024, Nesterenko *et al.* 2025). These applications typically rely on training models using multiple simulated datasets, further emphasizing the critical need for efficient sequence simulators.

Various sequence simulators have been previously developed (Cartwright 2005, Fletcher and Yang 2009, Dalquen *et al.* 2012, Bouchard-Côté and Jordan 2013, De Maio *et al.* 2022, Ly-Trong *et al.* 2022). These available tools differ in the types of data they can simulate (DNA, amino acids, codons), the substitution models they implement, and the indel length distributions they allow. For indel management, these tools typically implement the Gillespie algorithm (Gillespie 1977), which is described below. In most cases, substitutions and indels are generated simultaneously, while the algorithm for generating the true MSA is often not described (see below).

In this work, we present novel algorithms for simulating indels along a phylogenetic tree. We first provide a detailed description of how indels are simulated using a naive approach. We then describe two alternative approaches that simulate indels using specific data structures for tracking indel events. These data structures lead to a substantial decrease in running times. We also provide a detailed explanation of how true MSAs are computed following these simulations. Finally, we demonstrate the utility of our method by studying indel dynamics within *Chiroptera* protein MSAs.

## 2 Materials and methods

To lay the foundations for our new algorithms, we start by describing the basic algorithm, known as Gillespie algorithm, used to simulate indel evolution along a branch of a phylogenetic tree (Gillespie 1977). The common model used to simulate indel evolution assumes that indel rates and sizes are independent from the sequence content and that the indel rate scales linearly with sequence size. The indel location is distributed uniformly throughout the sequence and its size is drawn from a single distribution. As indel events change the sequence length, indel probability changes accordingly after each event. For a sequence of size *n*, there are *n *+ 1 potential insertion locations, which consist of the *n − *1 potential “spaces” between every two adjacent positions and both edges of the sequence. Let $r_{\mathrm{ins}}$ be the insertion rate. The sequence-wise insertion rate is ${R_{\mathrm{ins}}=(n+1)r}_{\mathrm{ins}}$. This sequence-wise insertion rate dictates the waiting time distribution for an insertion event: it is assumed that the time until the next insertion event is exponentially distributed with mean $1/R_{\mathrm{ins}}$. Note that “time” here is measured in units of number of substitutions per site, so when simulating along a branch, the initial time is 0, and the final time is the length of the branch. If an insertion event occurs, the event location is drawn uniformly from the available *n + *1 locations. The size of the inserted sequence is drawn from a pre-specified length distribution. Several distributions have been previously suggested to model indel sizes, the most common ones being the truncated versions of the geometric and Zipfian distributions (Benner *et al.* 1993, Lunter 2007, Wygoda *et al.* 2024). Content for the inserted positions is drawn from the stationary distribution of the substitution model, e.g., a JTT model (Jones *et al.* 1992) for protein sequences. Note that after each indel event, the insertion rate $R_{\mathrm{ins}}$ must be updated to account for the new sequence length.

Simulating deletion events is similar to insertion events with respect to deletion size and the need to update the sequence-wise deletion rate after each indel event. For deletion of size *k* that starts at position *i*, the deleted positions are *i*, *i *+ 1, …, *i *+* k − *1. In cases where the deletion size overflows (*i *+* k *− 1 > *n*), that is, there are not enough positions after the start position to accommodate the deletion, the deletion size is trimmed to the maximal available size. However, special care is needed to ensure that every position has the same probability of being deleted (Cartwright 2005). If the starting position of a deletion event was drawn uniformly in the range of 1 to *n*, the first position would only be deleted if an indel event starts at that position. In contrast, the second position in the sequence can be deleted by indel events starting at the second position, as well as indel events of length larger than one starting at the first position. The third position can be deleted by events starting at positions 1, 2, and 3. Thus, allowing deletion events to start uniformly along the sequence generates a bias against deletions at the beginning of the sequence.

### 2.1 Correcting for edge effects when simulating deletions along a branch

To account for deletion events that overflow into the sequence, an adjustment must be introduced. Given that a deletion event has occurred, we first draw its length $S_{d\mathrm{el}}$. An event of length $S_{\mathrm{Del}}$ can affect the sequence if it starts within the sequence or up to $S_{d\mathrm{el}}-1$ positions before the start of the sequence. We thus next draw the start location of the deletion event uniformly from $n+(S_{d\mathrm{el}}-1)$ potential locations. Thus, $R_{\mathrm{del}}=r_{\mathrm{del}}[n+(S_{d\mathrm{el}}-1)]$. We note that the effective deletion size, i.e., the number of deleted positions in the simulated sequence, is smaller than $S_{d\mathrm{el}}$ if the deletion starts before the actual sequence or ends after the end of the sequence. Yet, with this adjustment, assuming a deletion event of size $S_{d\mathrm{el}}$ has occurred, every position within the sequence has the same probability of being deleted, which is $S_{d\mathrm{el}}/(n+S_{d\mathrm{el}}-1)$. Note that for single character deletions ($S_{d\mathrm{el}}=1$), the probability of each position being deleted is exactly 1/n.

The waiting time until the next event (either insertion or deletion) follows an exponential distribution with parameter $\lambda =R_{\mathrm{ins}}+R_{\mathrm{del}}$, where $R_{\mathrm{ins}}$ and $R_{\mathrm{del}}$ are the sequence-wise rates of insertion and deletion events, respectively. Once an event occurs, it is classified as an insertion with a probability of $R_{\mathrm{ins}}/\lambda$ and as a deletion with a probability of $R_{\mathrm{del}}/\lambda$. This process continues iteratively, generating events and updating sequence lengths, until the remaining branch length is exhausted.

### 2.2 From simulating along a branch to simulations along a tree

To simulate sequence evolution along a whole tree, we start by generating the sequence at the root of the tree. Given a sequence size and a substitution model, we generate the root sequence and fill the positions using the stationary distribution of the supplied model. Then, given an ancestor sequence, we simulate each of its immediate descendant sequences along their corresponding branches. We repeat this procedure until all extant sequences are generated.

### 2.3 The complexity of the Gillespie algorithm

The Gillespie algorithm involves updating the sequence after each evolutionary event. In this algorithm, the evolving sequence is represented as an array or a list of characters. In such a representation, the cost of updating the sequence following an indel is O(n), where *n* is the current sequence length. For example, if the sequence is represented as an array, each indel requires copying O(n) elements. Let *k* denote the number of indel events that have occurred along the branch. In the worst case, these are all insertions, and the total sequence length after *k* insertions of maximal size *M* becomes n+Mk. We assume that *M* is small and fixed, and thus the cost of each indel event is O(n+k). As there are *k* such events, the total time complexity of the Gillespie algorithm for simulating indels along a specific branch is O(k(n+k)). We note that when evolution is simulated along long branches, *k* can be on the same order of magnitude as *n*.

### 2.4 Separation of substitution and indel simulation

**Figure 1.**
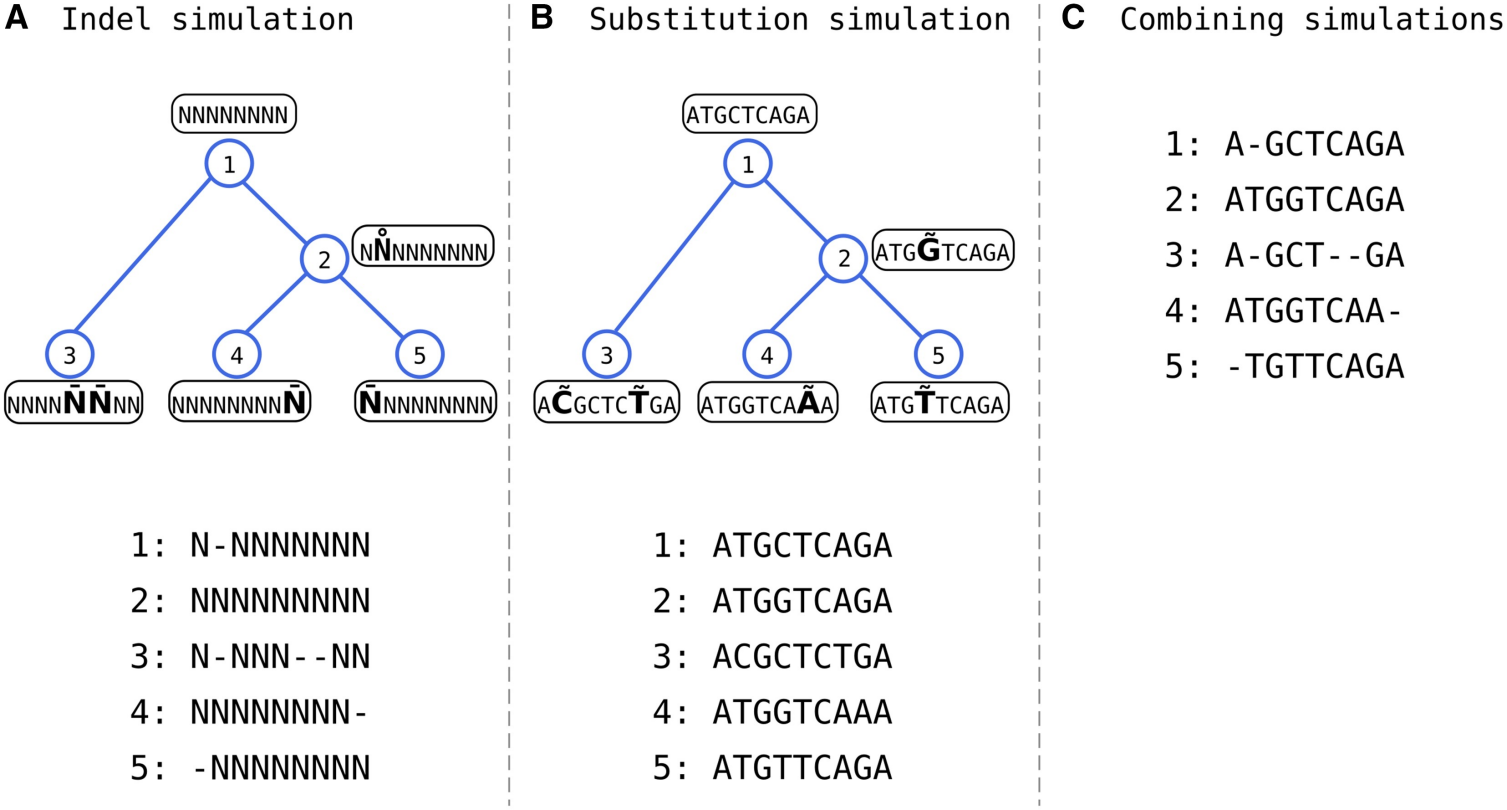
Separation of indel and substitution simulations and their merger. (A) Simulation of indel events while disregarding the sequence content, creating a template for the resulting alignment. (B) The resulting alignment size (in our example, the alignment size is 9) is used to simulate the sequence content and substitution events. (C) The indel template and sequence content are combined for the final alignment. Note that the alignments include both extant and ancestral sequences. Insertion and deletion events are marked with a small circle and line above the affected character, respectively. Substitution events are marked with a tilde symbol above the affected character.

We assume that the rate, size, and location of indels are not affected by substitutions and *vice versa*: the substitution type is indel-independent. This allows the separation of the simulation procedure into two independent processes. This separation can be achieved as follows. First, starting from an ancestral sequence, only indel events are simulated, without any substitutions. Figure 1A illustrates a resulting alignment in which all characters are marked with the letter “N”. This process determines the alignment length *L*. We then simulate substitutions by first drawing a random root sequence of length *L* and only simulating substitutions along the tree, i.e., disallowing indels to occur (Fig. 1B). The final simulated MSA is generated by superimposing the indel-only and substitution-only MSAs (Fig. 1C).

This simulation scheme has several advantages. First, it allows generating MSAs in which the substitutions are simulated and the indels are taken from empirical datasets, and *vice versa*, as was done in Trost *et al.* (2024). Second, it enables the introduction of specific accelerations to each module independently. Finally, our ABC-based approach for estimating indel model parameters from empirical MSAs relies on repeated simulations of the indel process. Substitutions are not needed for inferring the indel parameters, and thus, only the indel-only simulation model is used (Karin *et al.* 2017).

### 2.5 Faster indel simulation on a branch

We introduce a novel bookkeeping method that tracks events along the branch, allowing us to update the sequence only once after simulating the entire set of events that occurred along the branch. This new algorithm improves on the above O(k(n+k)) time complexity algorithm. The intuition behind our approach is straightforward. Consider an insertion event at a specific position. Since we simulate indels and substitutions separately, we can defer determining the inserted sequence until the end of the simulation. We only need to record the insertion’s length and starting position relative to the original sequence. If additional insertions occur within this segment, we simply update the inserted length. Similarly, deletions within the inserted segment only require updating the segment’s size. For a given branch, we only need to record which positions from the original sequence remain and the sizes of inserted sequences between them. Special handling is required for deletions that span both previously inserted segments and positions from the predecessor sequence.

### 2.6 Blocks

To allow for effective event tracking when simulating along a branch, we introduce the concept of blocks. Consider a sequence evolving along a branch of length *t*, where the starting sequence is labeled *s*. As a sequence evolves along this branch, it undergoes indel events, which are recorded using the block structure. The information is recorded relative to *s*. Because one must take special care of indels that can occur before the first sequence position, the actual positions within the sequence are numbered starting from one. We add a virtual anchor position, marked 0, at the beginning of the sequence. An insertion before position 1 will be included in the block that starts at position 0. We note that “position 0” is only used to keep track of events that occur to the left of the first position, and is not a real position.

A block is composed of two parts, the original part (OP) and the added part (AP). An OP consists of contiguous positions from the predecessor sequence, that were undisturbed by insertion and deletion events. The AP comprises positions that were added during the evolution simulated along the branch in question. Each block is represented by the 3-tuple (*start*, *length*, *insertion*), where the *start* is the position in the starting sequence where the OP starts, *length* is the length of the OP, i.e., the OP contains the position range [*start*, *start *+* length*] from the predecessor sequence, and *insertion* is the size of the AP (Fig. S1, available as supplementary data at *Bioinformatics* online). We note that while the AP can be of size 0, that is, *insertion *= 0, the OP size is assumed to be positive for all blocks.

We first demonstrate how the list of blocks is generated and updated during the process of evolution along a branch. We start with a sequence labeled *s* of length 100 characters (the corresponding data structure is shown in Table 1A). Accordingly, the block structure representing the initial sequence consists of a single block that starts at position 0, has a length of 101 (because of the inclusion of position 0 in the counting), and includes no insertions. This block is compactly written as (0, 101, 0), where the first index is the start position of the block relative to *s*, the second index is the length of the block, and the third index is the length of the inserted sequence.

**Table 1.** Example of the processing of the data structure used for bookkeeping indel events along a branch of a phylogenetic tree.^a^

A. Initial data structure		
Start	Length	Insertion
0	101	0

B. Following deletion of positions 80–84		
Start	Length	Insertion
0	80	0
85	16	0

C. Following insertion of size 5 after position 29		
Start	Length	Insertion
0	30	5
30	50	0
85	16	0

a Each row corresponds to a block. The events presented are processed sequentially: A → B → C.

Consider a branch with two indel events (Table 1). The first event is a deletion of size five, that occurred at position 80, i.e., positions 80–84 are deleted. The resulting sequence has two stretches of characters that match sequences in *s*. This can be compactly represented by splitting the original block into two. The first block starts at position 0 and has a length of 80 (again because position 0 is counted in the first block), and the second block starts at position 85 and has a length of 16 (positions 85–100, including both positions 85 and 100). The updated block list is shown in Table 1B. The next event is an insertion event of size 5 that started after position 29 (Table 1C). This event breaks the first block in two. The first block is now represented as (0, 30, 5), indicating that starting at position 0, the first 30 characters match sequence *s*, after which an insertion of five characters has occurred. The next block is represented as (30, 50, 0), indicating that starting from position 30, the following 50 characters match sequence *s*, with no following insertion. Of note, all blocks after the event remain unchanged, which directly contributes to the efficiency of the proposed algorithm. In our example, only the final block (85, 16, 0) remains unchanged. A more formal definition of a block and a detailed example are provided in Supplemental Information S1, available as supplementary data at *Bioinformatics* online.

### 2.7 Identifying the affected blocks

Updating the block list according to a new event necessitates identifying and updating all the blocks that are affected by the event. For brevity, when discussing the block list henceforth, we will use *S_i_*, *L_i_*, and *I_i_* for the start, length, and insertion of block *i*, respectively. Assume that the current block list is [(*S*_1_, *L*_1_, *I*_1_), (*S*_2_, *L*_2_, *I*_2_), …, (*S_k_*, *L_k_*, *I_k_*)]. Consider an indel event that occurred at position *q* relative to the current sequence. The total size of the first block is *T*_1_ =* L*_1_ +* I*_1_. If *q *>* T*_1_, then clearly the first block is not affected, and we can examine whether the event affects the second block. Similarly, if *q *>* T*_1_ +* T*_2_, the second block is not affected. By iteratively scanning the block list, we can identify the first block that is affected. Note that an insertion affects only a single block, whereas a deletion event may affect several consecutive blocks. Once the affected blocks have been identified, the block list must be updated.

**Figure 2.**
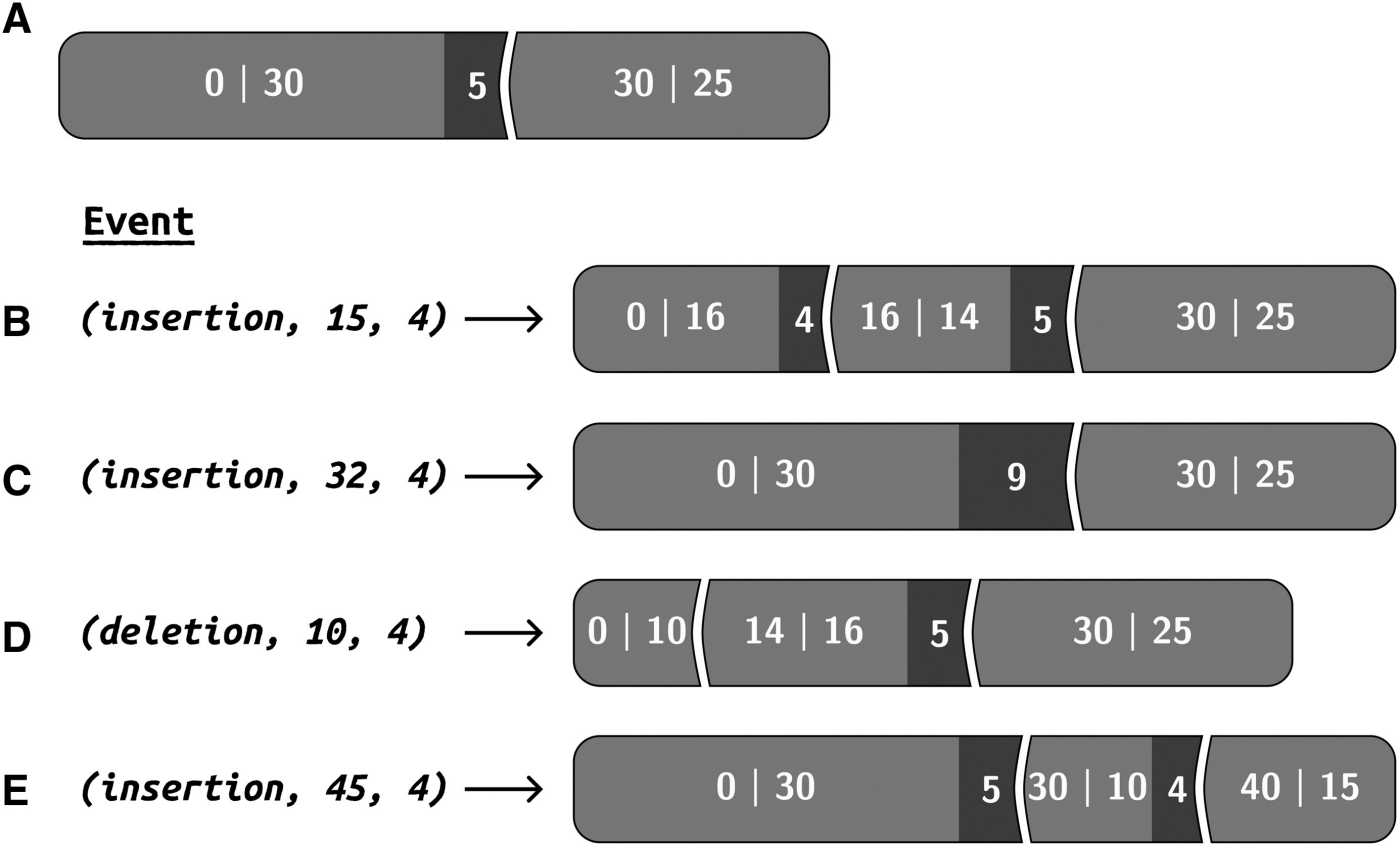
Examples of updating the block list structure following different indel events. Each block is represented as [start, length] with insertion length shown in dark boxes. (A) Initial block list with blocks (0, 30, 5) and (30, 25, 0). (B–E) Results of different indel events applied to the initial block list in (A): (B) Insertion event *(insertion, 15, 4)* occurring within the original part (OP) of the first block, splitting it into (0, 16, 4) and (16, 14, 5). (C) Insertion event *(insertion, 32, 4)* occurring within the added part (AP) of the first block, simply increasing the insertion length to 9. (D) Deletion event *(deletion, 10, 4)* removing positions 10–13 from the OP, splitting the first block into (0, 10, 0) and (14, 16, 5). (E) Insertion event *(insertion, 45, 4)* occurring in the second block’s OP, splitting it into (30, 10, 4) and (40, 15, 0).

To demonstrate this process of identifying the affected blocks and updating the block list, consider the starting block list [(0, 30, 5), (30, 25, 0)] illustrated in Fig. 2. Consider an insertion event of four characters that occurred after position 15 in the current sequence. Here, *q *= 15 and *T*_1_ = 35; since *q *<* T*_1_, we determine that the first block requires an update. The event occurred in the middle of the OP, i.e. it disturbed the segment that corresponds to the original sequence *s*. The block list is updated such that the first block becomes (0, 16, 4) and a new block (16, 14, 5) is added, as illustrated in Fig. 2B. Another example (Fig. 2C) starts with the same block list as Fig. 2A, but with event *(insertion, 32, 4)*. The event again affected the first block. Unlike the previous example, this event occurred within the AP rather than the OP. We simply update the AP size to accommodate the insertion, yielding block (0, 30, 9). Yet another example (Fig. 2D) starts with the same block list and event *(deletion, 10, 4)*. This deleted positions 10–13 from the predecessor sequence. The event affected the first block, splitting it into blocks (0, 10, 0) and (14, 16, 5). As a final example (Fig. 2E), we update the block list following the event *(insertion, 45, 4)*. Here, *q* = 45, exceeds the total size of the first block *T*_1_ =* L*_1_ +* I*_1_ = 35, which means that it did not affect it. Moving to the second block, we adjust *q* to represent the event position relative to this block: *q ← q* − 35 = 10. We can now treat the second block as if it is the first block and perform the same check again: *q = *10 is less than *T*_2_ = 25. This event thus affects the OP of the current block and is treated as in the first example, resulting in adjusting the second block to (30, 10, 4) and adding a new block (40, 15, 0).

More formally, the algorithm for locating affected blocks works as follows: given an event and a block list, we start by locating the first block that is affected by the event. To this end, we iterate over the block list and for each block *j* = (*S_j_*, *L_j_*, *I_j_*), we compare the total block length (*T_j_* = *L_j_* + *I_j_*) to *q*. If *q* > *T_j_*, we move to the next block and update *q* *←* *q* − *T_j_*. We continue until we find *j* for which *q* < *T_j_*. The full details of the algorithm for updating the block list are provided in Supplemental Information S2, available as supplementary data at *Bioinformatics* online.

### 2.8 Complexity of the block list structure approach

Let n denote the length of the starting sequence and assume that k is the number of indel events that occurred along the branch. Let b denote the size of the block list and n′, the maximal length of the sequence as it evolves along the branch. We first note that b≤min(k+1,n+1), since each event cannot add more than one block, and the worst-case scenario would be a block list with one block for each position in the predecessor sequence. Simulation of k events along a branch requires processing all k events. Each event has a time complexity of O(b), as we need to scan the block list linearly to find the location of the event and add or remove a block if needed. Therefore, the total time complexity for handling the events along a branch is O(kb). In contrast, the naive simulation approach has a time complexity of O(kn′), as each event requires an array reallocation for the sequence. When the sequences are long compared to the number of events, such that *b ≪ n*, the block list data structure approach should allow simulating indel events significantly faster than the naive approach.

We note that the number of blocks that are kept corresponds to the number of characters that remain from the ancestral sequence. Thus, after many deletion events have occurred, many of these ancestral positions would be deleted, resulting in fewer blocks. This suggests that at the beginning of the simulation, there is a single block. Following deletion events, this block is split, causing the number of blocks to initially increase. However, as additional deletion events occur, residues that correspond to the ancestral sequence are lost, thereby reducing the number of blocks.

### 2.9 Tree-based bookkeeping

When using a list to store the blocks, a linear search is conducted to find the block(s) affected by each event, resulting in O(b) operations. The AVL tree data structure (Cormen *et al.* 2009), which is a type of balanced binary search tree, allows more efficient searching, adding, and deleting of blocks, specifically in O(log(b)) operations. In our implementation, one AVL tree is associated with each branch of the tree topology. See Supplemental Information S3, available as supplementary data at *Bioinformatics* online, for a detailed description of the AVL-based block tree data structure.

### 2.10 Generating the MSA from a set of simulated sequences

**Figure 3.**
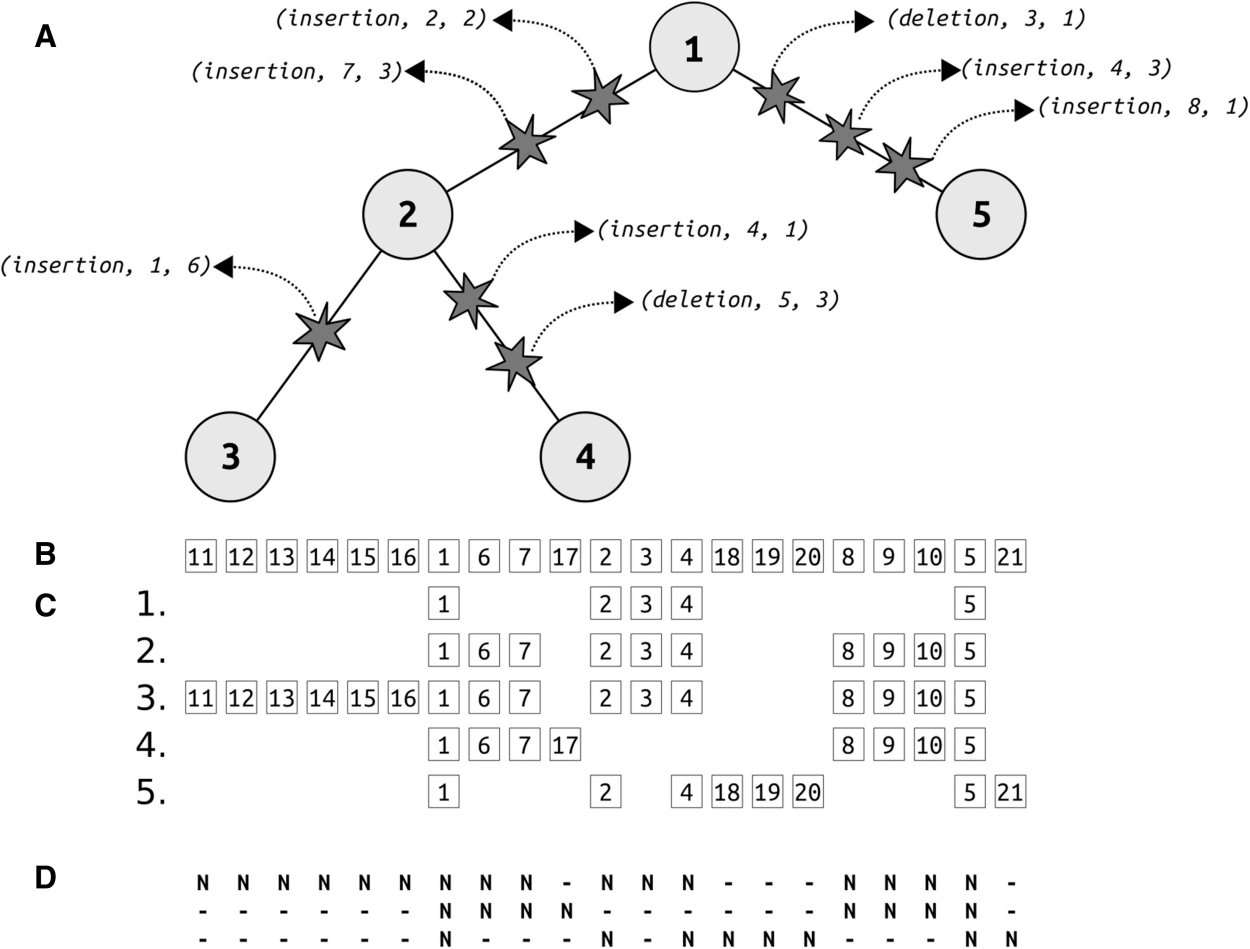
Evolution of the sequence alignment through indel events. (A) Phylogenetic tree showing three species and five nodes (numbered 1–5) with indel events (represented by stars) along the branches. Each event is labeled with its type, position, and length. (B) The final super-sequence after preorder tree traversal. The super-sequence is updated following insertion events, with each insertion introducing new characters (numbers) to the super-sequence. In the above example, the insertions along the branch from node 1 to node 2 introduced numbers 6–7 and 8–10. (C) Alongside updating the super-sequence, the sequences at each node are updated, this time also accounting for deletions. Each sequence is updated relative to its predecessor sequence. The initial root sequence (Row 1) contained five characters (positions 1–5). Rows 1–5 show the specific subsequence associated with each node in the tree. Aligning each sequence with the super-sequence is trivial because each position in the super-sequence defines a column in the final alignment. (D) The final template alignment.

Along with the simulation processes described above, it is possible to generate the “true” MSA underlying the evolving sequences, which depends on the ancestral sequence and the complete history of evolutionary events. To achieve this, we implement a “super-sequence”, a linked-list data structure that integrates the root sequence with all insertions that occurred throughout the simulation. Figure 3 demonstrates how the super-sequence is used to reconstruct the true alignment by tracking the details of events along each branch. The super-sequence serves as a comprehensive template that preserves positional information across all sequences by tracking the exact locations of insertions. During simulation, each node in the phylogenetic tree maintains a current list of references to specific positions in the super-sequence. This list is called a “pointer sequence” because each element in the list refers to a position in the super-sequence. To generate the final MSA, we extract the pointer sequences from the leaf nodes and align them according to their references to the super-sequence, with each referenced position corresponding to a column in the alignment. Importantly, some positions in the super-sequence may represent characters that were deleted in all extant sequences. Therefore, each node in the super-sequence contains a “column” flag indicating whether that position is referenced by any leaf pointer sequence. This prevents the inclusion of gap-only columns in the final alignment, ensuring that the MSA accurately reflects only the evolutionary relationships observable in the leaf sequences. A more detailed description, including how the true MSA is generated using the block structure, is provided in Supplemental Information S4, available as supplementary data at *Bioinformatics* online.

### 2.11 Complexity of indel-only MSA generation

For each event, the generation of the pointer sequence has a time complexity of O(n′). Given *k* events along a branch, the total time complexity of the naive algorithm is O(kn′). When using the block list structure, there is only a single pointer sequence that is generated along a given branch (see Supplemental information S4, available as supplementary data at *Bioinformatics* online), and thus the total time complexity for updating the pointer sequence is O(n′), while the total time complexity of the algorithm is O(kb+n′). When using the tree-based bookkeeping, the complexity is reduced to O(k⋅log(b)+n′). These factors must be summed over all tree branches to obtain the complexity of the entire simulation process.

### 2.12 Comparing different simulation methods

We first compared the runtime of three different methods for indel simulation along a phylogenetic tree: (i) Naive, in which the simulator applies each event directly on a copy of the parent sequence as it occurs along the branch of the phylogenetic tree. Intuitively, simulating in this manner should be significantly slower than the bookkeeping approach described above; (ii) Block list, which maintains a condensed log of events occurring along each branch; and (iii) Block tree, which uses a balanced binary tree to store the blocks. All algorithm implementations were coded in Python 3 and are available at: https://github.com/nimrodSerokTAU/evo-sim.

### 2.13 Benchmarking setup

In this comparison, the rates for insertions and deletions were set to 0.03 and 0.09, respectively, reflecting values within the range of empirical datasets (Graur *et al.* 1989, Cartwright 2009). A Zipf distribution, truncated at 50, was assumed to model the length distribution of indels (Fletcher and Yang 2009). The Zipf parameter was set to 2.0, which translates to a mean length of 2.76 characters for both insertions and deletions (Loewenthal *et al.* 2021). A total of 541 trees were taken from the OrthoMaM v8 database (Douzery *et al.* 2014), all trees contained 40 species. The average sum of all branch lengths per tree was 2.96 ± 0.95 substitutions per site. The average branch length across all the trees was 0.038 ± 0.059 substitutions per site. All trees are available in the GitHub repository “evo-sim” under the “benchmark/TRUE_TREES” directory. Of note, when comparing the naive, block list, and block tree simulators, we used the exact same events for each method, and the differences in performance thus reflect only the handling of these events.

### 2.14 The contribution of indels versus substitutions to running times

We tested under which conditions indel simulation constitutes the computational bottleneck in a simulation of sequence evolution. To this end, we also implemented a substitution simulator (in Python) for the JTT amino acid replacement model. In this simulator, amino acid replacement probabilities are computed using the probability matrix approach (Ly-Trong *et al.* 2022), by exponentiating the rate matrix *Q* using eigen-value decomposition. These transition probabilities are precomputed for each branch of the phylogenetic tree. Substitutions are then determined based on these values for each site and for each branch. As stated above, substitutions and indels are computed independently and the resulting MSA is then obtained by superimposing the results of the two simulators. The cost of indels versus substitutions in running times was next calculated across varying sequence lengths (100, 500, 1000, and 5000 characters) and branch lengths (0.01, 0.05, 0.1, and 0.5 substitutions per site) using a representative tree from the OrthoMaM dataset (“AATF_true_tree.txt” in the “benchmark/TRUE_TREES” directory) with all branches normalized to the aforementioned lengths. Using the same indel rates as above (0.03 insertion, 0.09 deletion), we measured the relative computational time spent on indel versus substitution simulation.

### 2.15 The effect of insertion to deletion rate ratio and the tree divergence on running times

To further understand the runtime differences between the block list and block tree structures, we compared the MSA simulation performance for each data structure under the following simulation settings: varying insertion rates from 0.01 to 0.09 in increments of 0.01, while maintaining a total indel rate of 0.1 (e.g., insertion rate 0.02 with deletion rate 0.08). This analysis was repeated across three different branch length scaling factors (1, 5, 10) and used a representative tree from the OrthoMaM dataset with all branches normalized to 0.1.

### 2.16 Comparison with AliSim

In addition to this internal benchmarking, we compared the performance and memory usage of the block tree algorithm with AliSim (Ly-Trong *et al.* 2022), the current state-of-the-art in MSA simulations. The simulators were compared on the same setup described in the previous paragraph, once with substitutions and once without substitutions (indel-only simulation). To prevent AliSim from computing substitutions, we set the invariant sites proportion to 0.999999, effectively eliminating substitutions.

### 2.17 Empirical data analysis setup

In this analysis, we considered protein MSAs from the OrthoMaM v12 database (Allio *et al.* 2024). The data curation was based upon two criteria: (i) each MSA contained at least 16 species from the order *Chiroptera*; and (ii) the MSA included at least 14 different (unaligned) sequence lengths. These criteria ensured sufficient indel data within these datasets and resulted in 47 protein MSAs. Following this, we split each MSA into two separate alignments: one containing only chiropterans and the other containing all remaining mammals. We then computed the corresponding tree for each of the MSAs using the RaxML-NG tree search software (Kozlov *et al.* 2019) with the WAG + G4 substitution model. Finally, we ran the SpartaABC inference on each of the datasets to infer the indel model parameters. Note that we assumed the length distribution of both insertions and deletions follows a truncated Zipfian distribution, with a maximum indel length of 50.

## 3 Results

### 3.1 Benchmarking

**Figure 4.**
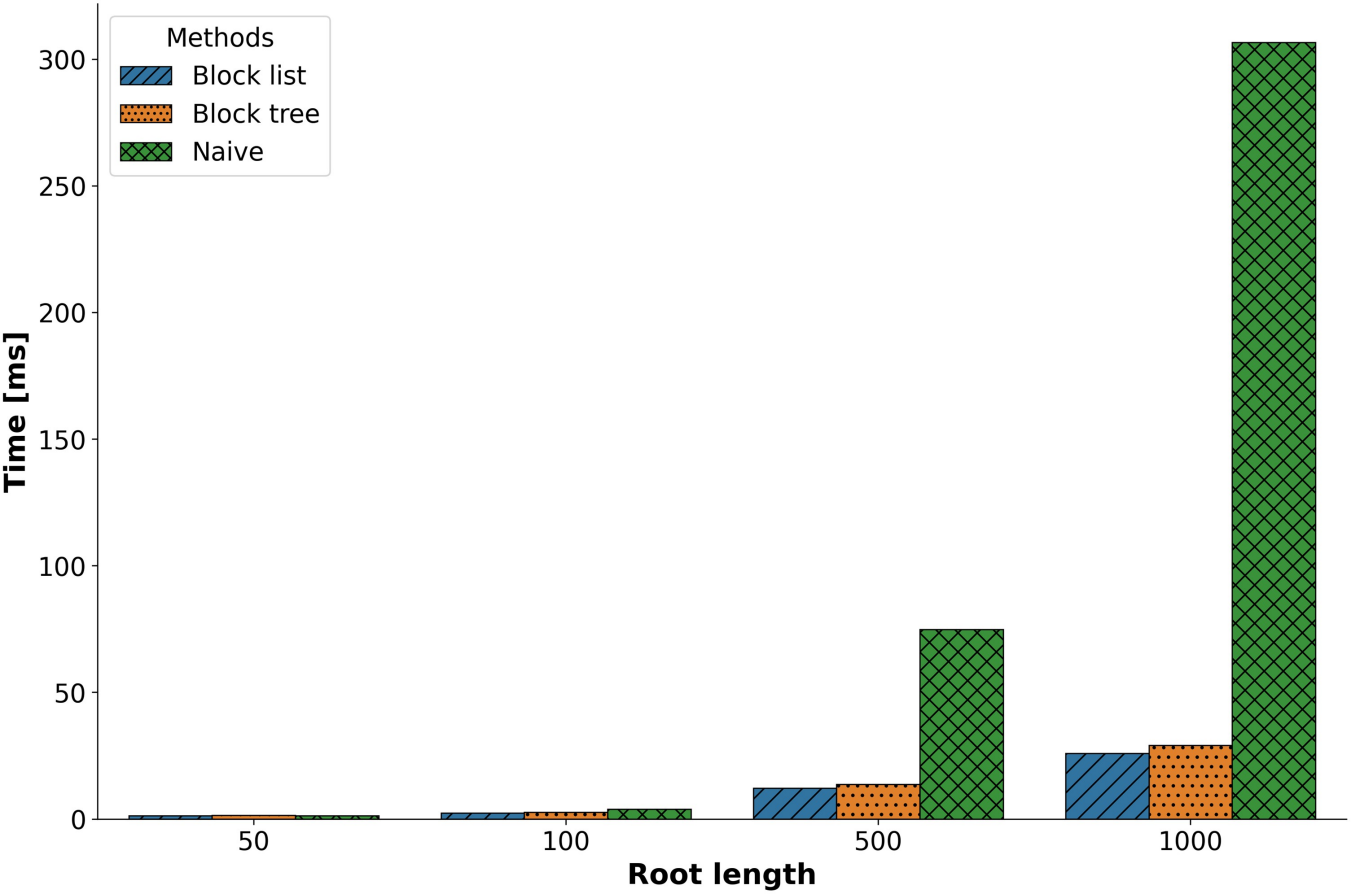
Runtime comparison of the different indel simulation methods on the 541 OrthoMaM trees, across different root length values.

We compared the performance of the three methods (naive, block list, and block tree) using four different root sequence lengths: 50, 100, 500, and 1000 characters. For this comparison, we only simulated indel events (without substitutions). We observed a substantial improvement in runtime when using either the block list or block tree methods compared to the naive implementation. These differences in runtime became more pronounced for longer sequences (Fig. 4). There was a significant difference in mean runtime between the simulation methods across the four different root lengths (two-way ANOVA; *P *<.05), with no significant difference between the block list and block tree methods (Tukey test; *P *<.05). Detailed running time values are provided in Table S1, available as supplementary data at *Bioinformatics* online.

**Figure 5.**
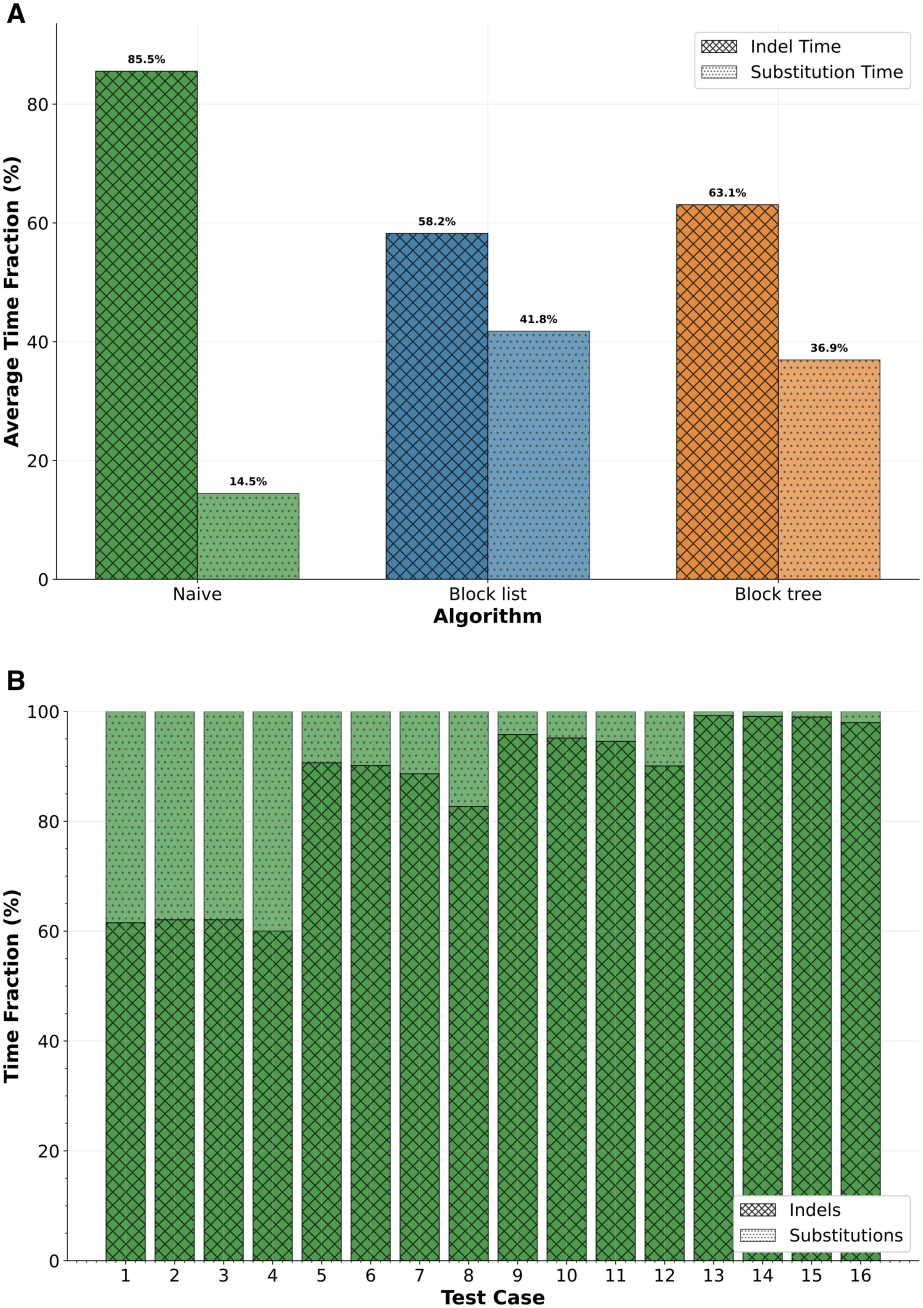
The relative runtime dedicated to the generation of indels versus substitutions when simulating MSAs. (A) The average time taken by indels versus substitutions in MSA simulation across all tested parameters. (B) Time fraction trends for naive indel simulation across the 16 test cases. Test cases 1–4: MSAs generated with a root length of 100 amino acids; test cases 5–8: Root lengths of 500 amino acids; test cases 9–12: root lengths of 1000 amino acids; test cases 13–16: root lengths of 5000 amino acids. Within each group, branch lengths varied in increasing order: 0.01, 0.05, 0.1, 0.5. For example, in test case 13 all branch lengths were 0.01 substitutions per site, and in test case 14, all branch lengths were 0.05 substitutions per site.

The above results demonstrate that the block list and block tree structures can substantially reduce runtime when simulating indel events. We next assessed the relative runtime dedicated to the generation of indels versus substitutions when simulating MSAs. On the benchmark dataset (see Section 2), the percentage of time spent on simulating indels was 85.5% on average for the naive method (Fig. 5A). The length of the root sequence affected the relative running times: when the length was short (100 characters), the percentage of time devoted to indels was less than 65%. For root sequences of 5000 characters, the percentage was more than 98% (Fig. 5B). When considering the block list and block tree structures, indel simulation took on average 58.2% and 63.1% of the total time, respectively, meaning both methods alleviated some of the computational burden from indels toward substitutions compared with the naive approach.

**Figure 6.**
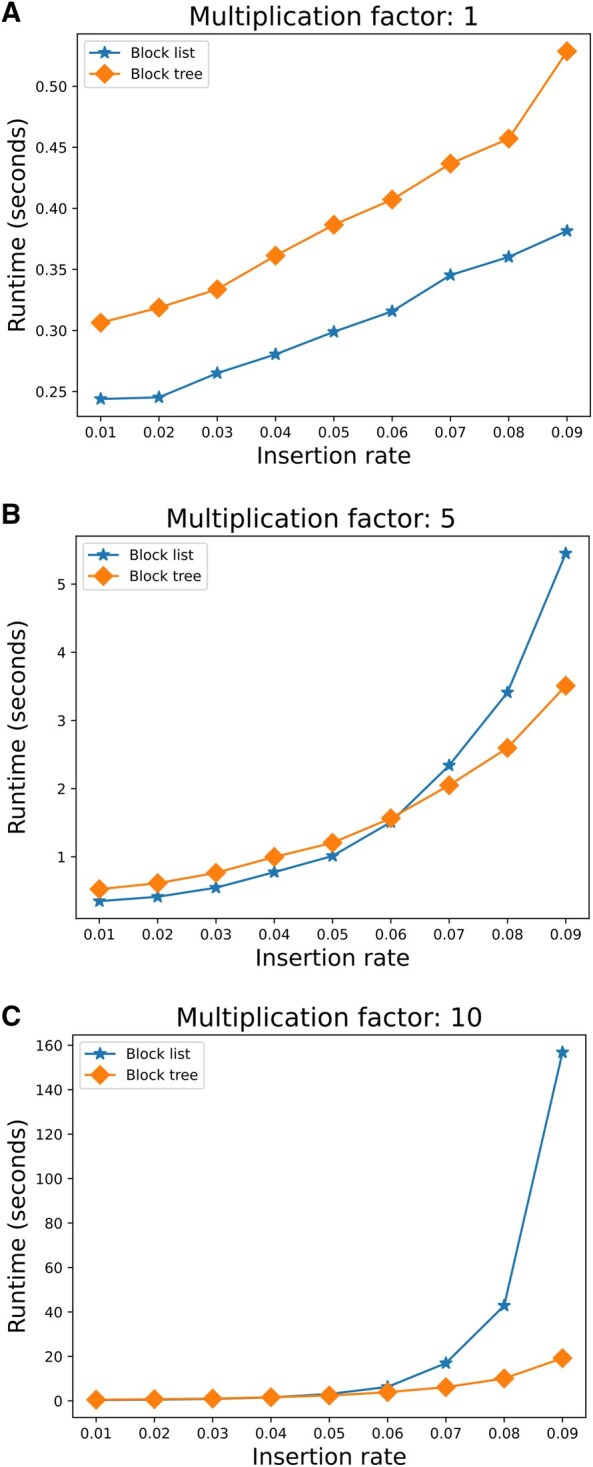
Performance comparison of the block list and block tree methods across different branch length multiplication factors and indel rates.

The similar runtime performance between the block list and the block tree partially reflects differences in implementation details (the block list implementation is based on Python lists, which are highly efficient). Moreover, the tree-based structure is expected to be much faster when the number of events per branch is large. We hypothesized that the number of events experienced in the above benchmark was not enough for these differences to become apparent. To test this hypothesis, we selected a tree from the above OrthoMaM dataset and set all the branch lengths within it to 0.1 substitutions per site. We then compared the running times of the block list and block tree algorithms when the branch lengths were multiplied by a factor of either 1, 5, or 10, and the root length was set to 10 000 characters. Additionally, we tested how different insertion-deletion ratios affect the runtime. Starting each time with an insertion rate of 0.01 and increasing it incrementally up to 0.09 while maintaining a constant sum of insertion and deletion rates of 0.1. For insertion rates above 0.06, increasing the branch length by a factor of 5, resulted in a small advantage (10%) in the performance of the block tree method. The difference was more pronounced when increasing the branch lengths by a factor of 10, e.g., when the insertion rate was 0.09, the block tree method was eight times faster than the block list method (Fig. 6). Thus, the block tree method is superior to the block list in some extreme settings, specifically with high insertion frequencies per branch. Even so, in trees originating from highly homologous sequences, the block list method offered a slightly better or similar performance overall when considering lower insertion rates.

### 3.2 Comparison with AliSim

To assess the performance and memory usage of our proposed block-based methods compared with existing MSA simulation programs, we ran AliSim (Ly-Trong *et al.* 2022) and our block tree-based simulator using the parameter sensitivity analysis setup described above (insertion rates 0.01–0.09, total indel rate 0.1, and branch length scaling factors 1, 5, and 10). We measured both peak memory usage and runtime for both indel-only simulations with both indels and substitutions.

**Figure 7.**
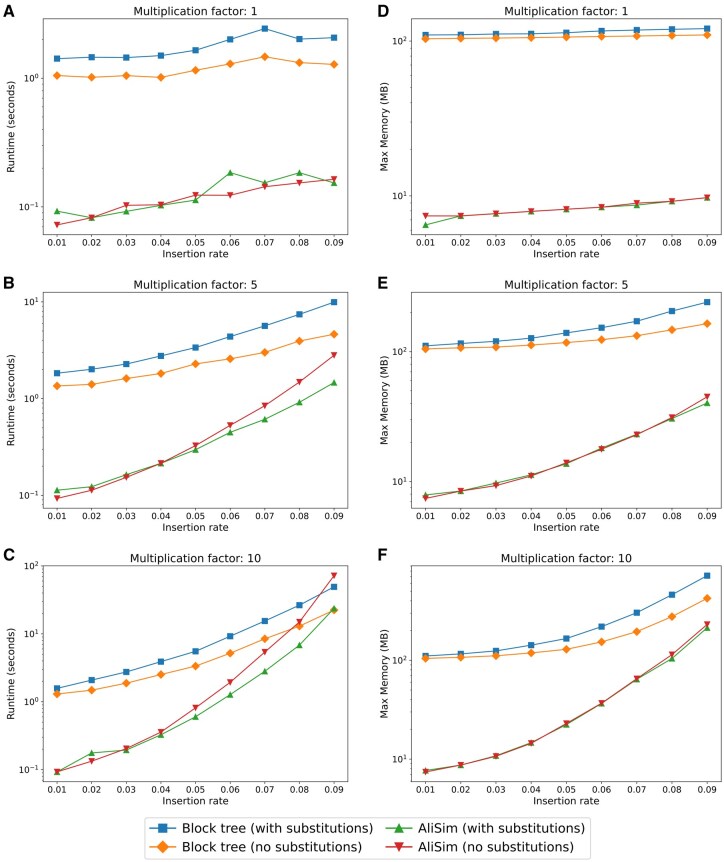
Performance comparison of the block list and block tree methods across different branch length multiplication factors and indel rates. (A–C) Runtime (seconds) for 1×, 5×, and 10× branch scaling factors. (D–F) Peak memory usage (MB) for the corresponding factors. squares: block tree with substitutions; diamonds: block tree without substitutions; triangles: AliSim with substitutions; inverted triangles: AliSim without substitutions.

As expected, given the implementation differences between AliSim’s highly optimized C++ codebase and our proof-of-concept Python implementation, AliSim generally achieved faster runtimes across most parameter combinations (Fig. 7). The performance gap narrowed considerably under extreme conditions, particularly at 0.09 insertion rate with a scaling factor of 10, where runtimes became comparable (Fig. 7F). Both simulators exhibited memory usage that scaled with indel complexity, with AliSim maintaining a peak consumption below 230 MB and our implementation starting from a higher baseline that peaked at 718 MB under extreme parameter combinations (Fig. 7C). The difference in baseline memory consumption primarily reflects the inherent overhead of Python compared to C++, while both implementations showed the expected increase in memory usage as indel rates and scaling factors increased. Notably, even at peak usage, our implementation’s memory requirements remain well within the capabilities of modern computing systems. Thus, even with the increase in memory usage, the corresponding gains in computational speed make our block-based approach a worthwhile addition to modern MSA simulators.

As mentioned above, AliSim is implemented in C++, while our simulator uses Python. Based on comprehensive benchmarking studies, Python implementations typically execute 8–29× slower than equivalent C++ code (Lion *et al.* 2022), suggesting our block-based approach could achieve competitive performance when implemented in a compiled language. This performance differential indicates that the algorithmic advantages of our method may be currently masked by language-level overhead.

### 3.3 Empirical data analysis

As a proof-of-concept, we integrated our bookkeeping approach within the SpartaABC framework to infer indel dynamics across mammals (Karin *et al.* 2017, Loewenthal *et al.* 2021). We specifically compared bats (order *Chiroptera*) with other mammals to investigate whether the known smaller genome size of bats could be explained by a higher deletion rate (Hughes and Hughes 1995).

We performed a two-sided Wilcoxon test on the inferred indel parameters (deletion rate, insertion rate, and length distributions) to compare chiropterans with other mammals. There was no significant difference in the insertion length parameter between groups (*P *> .05). The mean, standard deviation, and *P*-values of the indel model parameters across the two groups are summarized in Table 2. Surprisingly, chiropterans exhibited a higher insertion rate and a lower deletion rate compared to other mammals, as well as a higher average deletion length parameter, i.e., shorter deletions (Table 2). These results might discount indels as a plausible cause for their smaller genome size.

**Table 2.** Mean and standard deviation of the inferred indel parameters for the 47 datasets, alongside the *P*-values for the Wilcoxon test.

	Insertion rate	Deletion rate	Insertion length parameter	Deletion length parameter
Chiropterans	0.009 ± 0.003	0.029 ± 0.006	1.50 ± 0.09	1.20 ± 0.06
Other mammals	0.007 ± 0.002	0.035 ± 0.007	1.53 ± 0.07	1.11 ± 0.05
*P*-value	4.89 × 10^–5^	4.76 × 10^–7^	.09	7.07 × 10^–11^

As stated above, efficient simulations using the block tree algorithm were implemented within SpartaABC. The entire ABC-based computation of the above empirical data (MSAs of chiropterans and other mammals) took 1086.8 h, with 756.1 h dedicated to simulations. In comparison, we estimated that the naive approach would require 11 116 h (Supplemental Information S5, available as supplementary data at *Bioinformatics* online). This 14-fold speed improvement demonstrates the utility of our simulation algorithm, enabling ABC-based inference of indel parameters at a genomic scale.

## 4 Discussion

Sequence simulation is widely used in the study of gene evolution. In this work, we introduced two novel algorithms for the simulation of indel evolution along a tree. Both algorithms have lower time complexity compared to the naive method. In addition, we have shown that our bookkeeping methods for tracking indel events along a branch result in a significant speed-up compared to the naive approach.

Our proposed list-based approach requires iterating over the entire event history, which for extreme cases may still burden the simulation. In contrast, the AVL-based approach greatly reduces this burden. However, pointer-based data structures such as AVL trees may result in cache misses, which can significantly impact algorithm speed in real environments (Saikkonen and Soisalon-Soininen 2016). The real-time performance of the algorithms proposed here may vary depending on the computer language used and the implementation details of the utilized data structures. Other data structures used for text editing, such as rope (Boehm *et al.* 1995) or B-tree (Bayer and McCreight 1972), can also be considered to accelerate similar computations. Of note, binary search trees were recently proposed to expedite simulations with indels specifically for short branches with few events (De Maio *et al.* 2022).

Using the algorithms presented here, we accelerated SpartaABC, an ABC approach to infer indel dynamics (Loewenthal *et al.* 2021). We applied it to compare indel dynamics of bats versus other mammals. Our results suggest that indels may not be the causative evolutionary process that led to the small genome size observed in bats. However, additional analyses of non-coding regions are needed to further validate this conclusion. This type of analysis greatly benefits from the bookkeeping method, as it requires numerous simulations of indel-only MSAs with a wide range of indel parameters.

One of the most challenging aspects of simulation studies is to generate data that resemble empirical data. This is difficult because we usually do not fully understand the evolutionary dynamics that led to current-day sequences. Inferring indel dynamics that capture the patterns in a given dataset is particularly complex (Trost *et al.* 2024). The algorithms presented here, combined with SpartaABC and substitution-based models, enable efficient generation of multiple MSAs that match the indel evolutionary patterns observed in empirical data.

Many aspects of sequence evolution remain unaddressed in current indel simulation models. For instance, all these models assume that indel probability is independent of sequence context. However, clear evidence contradicts this assumption. Indel dependence on sequence context has been demonstrated at both the DNA level (Tanay and Siggia 2008, Kvikstad *et al.* 2009) and the amino acid level (Chang and Benner 2004, de la Chaux *et al.* 2007). Integrating context-dependent indel models into efficient simulation algorithms represents an important research frontier.

## Supplementary Material

btaf686_Supplementary_Data

## Data Availability

The data underlying this article are available at https://github.com/nimrodSerokTAU/evo-sim
