# Supplementary material for: Efficient algorithms for simulating sequences along a phylogenetic tree
Source: Bioinformatics. 2025 Dec 29;42(1):btaf686. doi: 10.1093/bioinformatics/btaf686 (PMC12797210; doi:10.1093/bioinformatics/btaf686)
Supplement: btaf686_Supplementary_Data [file btaf686_supplementary_data.pdf]

Efficient algorithms for simulating sequences along a phylogenetic tree

Supplemental Information

Elya Wygoda[1], Asher Moshe[1], Nimrod Serok[1], Edo Dotan[1,2], Noa Ecker[1], Naiel Jabareen[1], Omer Israeli[1], Itsik Pe'er[3], and Tal Pupko[1]†

[1]The Shmunis School of Biomedicine and Cancer Research, George S. Wise Faculty of Life Sciences, Tel Aviv University, Tel Aviv 69978, Israel.

[2]The Henry and Marilyn Taub Faculty of Computer Science, Technion – Israel Institute of Technology, Haifa 3200003, Israel.

[3] Department of Computer Science, Columbia University, New York, New York, USA.

† To whom correspondence should be addressed:

Tal Pupko; E-mail: talp@tauex.tau.ac.il

Keywords: simulations, evolutionary models, indels, alignment.

Supplemental Information S1: Bookkeeping data structure

The block list structure

(a)

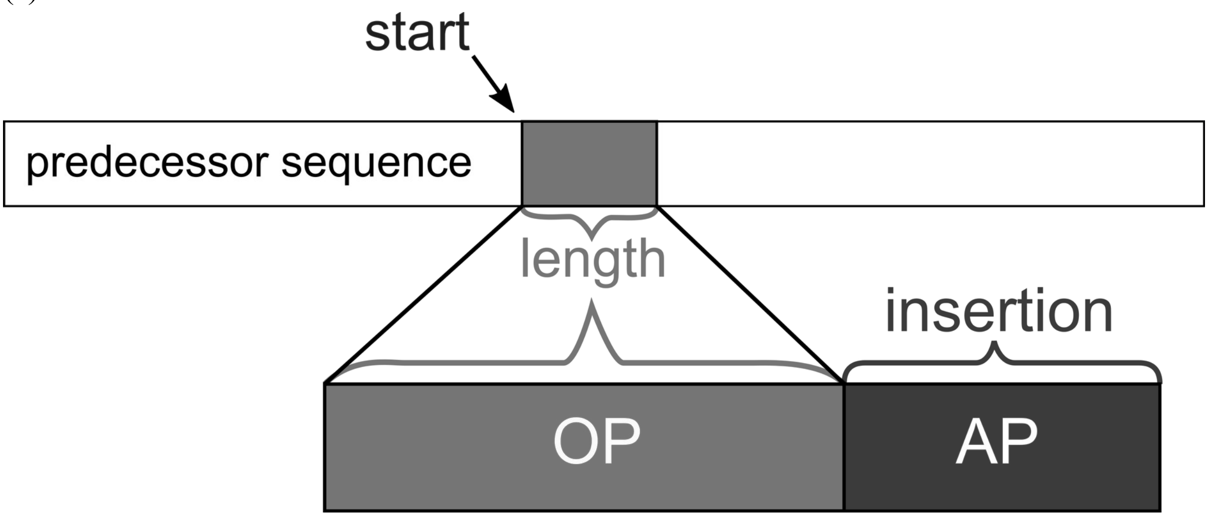

(b)

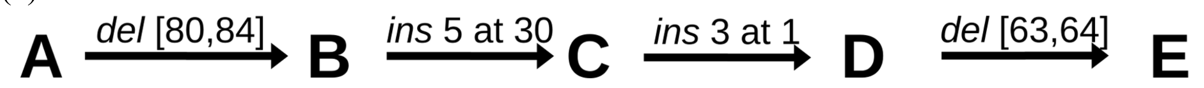

(c)

A.

Start	Length	Insertion
**0**	101	0

B.

Start	Length	Insertion
**0**	80	0
**85**	16	0

C.

Start	Length	Insertion
**0**	30	5
**30**	50	0
**85**	16	0

D.

Start	Length	Insertion
**0**	1	3
**1**	29	5
**30**	50	0
**85**	16	0

E.

Start	Length	Insertion
**0**	1	3
**1**	29	5
**30**	25	0
**57**	23	0
**85**	16	0

Figure S1. The block list structure. See text below for explanation.

To enable effective event tracking when simulating evolution along a branch, we introduce the concept of blocks. Each block consists of two parts: the original part (OP) and the added part (AP). The OP comprises contiguous positions from the predecessor sequence that remain undisturbed by insertion and deletion events. The AP consists of contiguous positions added during the evolutionary process along the branch. The AP corresponds to characters inserted to the right of the OP.

Each block is represented by the 3-tuple (start, length, insertion):

- start: the position in the predecessor sequence where the OP begins
- length: the length of the OP, containing the position range [start, start + length)
- insertion: the size of the AP

While the AP can have size 0 (insertion = 0), the OP size is always positive. When an OP is fully deleted, the block is removed and its AP is merged with the previous block.

A more elaborate example from that given in the main text. Consider the event C → D in S1b: an insertion of size three at the beginning of the sequence, that is, after position 0 (Figure S1cD). The last event, D → E, is a deletion of size two that starts in position 63 in the current sequence. Of note, this position corresponds to position 55 in the predecessor sequence, due to the insertion of a total of 8 characters to the left of the deletion point (see the next section for a detailed algorithmic description). As the record in the data structure is relative to the predecessor sequence, this means that positions 55-56 of the original sequence were deleted. Thus, the number that represents the starting point of the new block in the data structure is 57 (rather than 65), which yields the data structure shown in Figure S1cE. We track the total sequence size using a special attribute in the block list object, updated after each event.

Reconstructing sequence evolution from a list of blocks

We now explain how to reconstruct the resulting sequence from a list of blocks. Figure S1b illustrates the event history that led to the final list of blocks. The first block indicates an insertion of three characters before position 0. Note that this block has length one because it includes position 0. The second block shows that the next 29 characters (positions 1–29) come unchanged from the starting sequence, followed by a 5-character insertion after position 29. The third block takes 25 unchanged characters (positions 30–54). The fourth block starts at position 57 and takes 23 characters (positions 57–79). Since the last copied position was 54, this reveals that positions 55–56 were deleted. The final block starts at position 85, indicating additional deletions, and shows that positions 85–100 remain unchanged.

Supplemental Information S2: Handling block list events

Updating the block list following an insertion event

Assume an insertion event *(insertion, s, l)* that was found to occur on block j (and accordingly $q < T_j$). If the start location of the event is in the OP (i.e., $q < L_j$), we break the block into two blocks at q. Block j is updated to (S_j, q, l) and a new block $(S_j + q, L_j - q, I_j)$ is added after block j. Otherwise, the location is within the AP. In this case, there is no need to add a new block, and we simply add l (the size of the insertion event) to I_j. The updated block is (S_j, L_j, I_j+l).

Updating the block list following a deletion event

Unlike an insertion event, a deletion event can affect more than one block, i.e., it overflows to the next blocks. Let the deletion event be *(deletion, s, l)*. We start by describing a deletion event that is contained within a single block. In this case, $q + l \leq T_j$. We divide the handling of such events into different cases: (1) The deletion begins at $q = 0$ and removes the entire original part (Figure S2A) of the very first block. In this case, we retain the first block and update it to be (0, 1, p), where p is the part that remains from its AP component (p can be zero); (2) The deletion begins at $q = 0$ of a block, which is not the first, and removes the entire original part (also Figure S2A). In this case, we delete the block entirely and add the part that remains from the AP component to the AP of the previous block. Formally, we update block j - 1 to $(S_{(j-1)}, L_{(j-1)}, I_{(j-1)} + (T_j - l))$; (3) If the deletion starts at $q = 0$ but preserves part of the original segment (Figure S2B), we update block j to $(S_j + l, L_j - l, I_j)$. For deletions that start and end within the original part ($q > 0$) (Figure S2C), we split into two blocks: $(S_j, q, 0)$ before the deletion and $(S_j + q + l, L_j - q - l, I_j)$ after it. When a deletion starts in the original part but extends into the appended part (Figure S2D), we trim the block to $(S_j, q, I_j - (l - (L_j - q)))$. Finally, if the deletion affects only the appended part (Figure S2E), we simply reduce the insertion count, making block j become $(S_j, L_j, I_j - l)$.

For deletion events that overflow into the next block, we split the event (*deletion*, s, l) to $\dot{e}$ which is contained in the current block, $\dot{e}$=(*deletion*, s, $T_j - q$), and the event $\ddot{e}$ that is the rest of the event, $\ddot{e}$=(*deletion*, 0, $l - T_j - q$).

The implementation of the algorithm differs slightly from the high-level description above. We therefore supply a GitHub repository containing the actual implementation in the Python programming language.

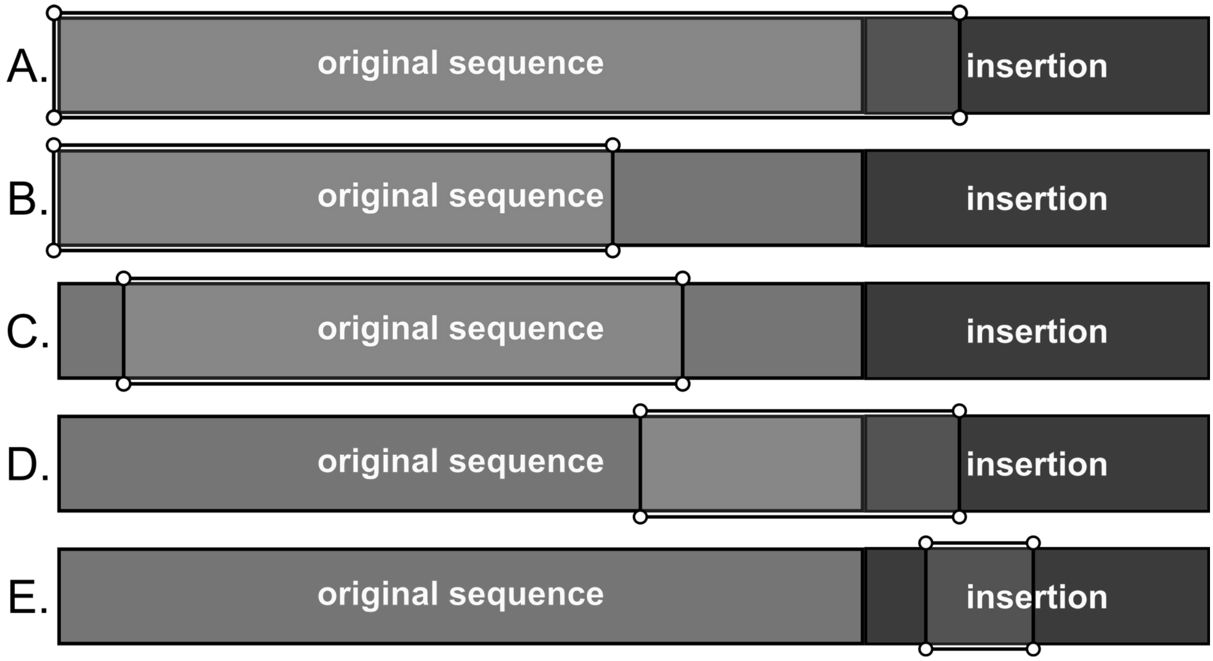

Figure S2. illustration of the different deletion scenarios. A. A case in which the entire OP is deleted. B. A case in which only the head of the OP is deleted. C. A case in which the middle of the OP is deleted, leaving parts of the OP before and after the event. D. A case in which the tail of the OP is deleted. E. A case in which the deletion occurs only in the AP.

Supplemental Information S3: The block tree data structure

The AVL tree structure

We next describe the implementation of the AVL-tree approach for a single branch. Each node within the AVL tree represents a block (as above, the block stores information about the contiguous positions from the predecessor sequence and regarding positions that were added during the evolution). Recall that for block i, S_i denotes the starting position relative to the predecessor sequence. The AVL is sorted so that the block with the lowest S_i value is the leftmost leaf, and the rightmost leaf in the AVL tree corresponds to the block with the highest S_i value. The S_i value in the root of the AVL tree usually corresponds to a position in the middle of the predecessor sequence. In other words, the AVL tree is sorted using a key, which is the S_i value.

In the block-list data structure above, following an indel event, two actions were applied: a search for the affected blocks and an update of the affected blocks. Using the AVL data structure, the search is more efficient, and the update remains the same. The block structure assigned to each AVL node is slightly different from the block structure in the block list. Specifically, we keep to additional attributes for each block: (1) a “size” attribute that holds the total size of all blocks within its subtree, including that block; (2) a “next” attribute that points to the node with the successor key, i.e., the next block relative to the predecessor sequence (Figure S3).

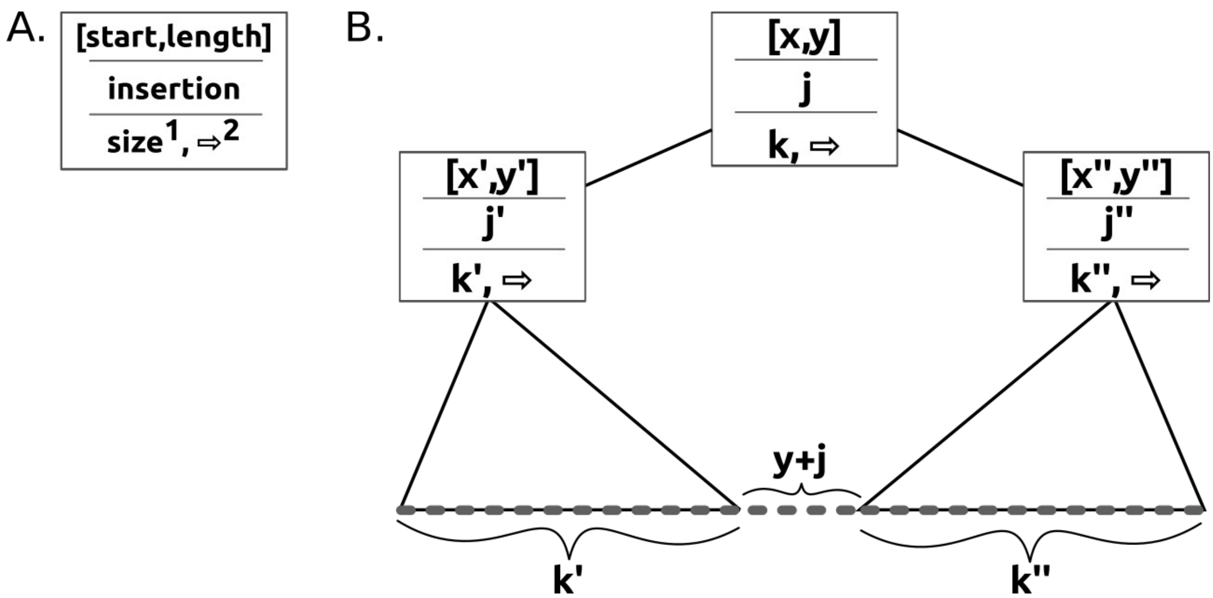

Figure S3: The anatomy of an AVL block tree. A. The AVL node, which contains two additional attributes compared to blocks in the block list: 1. size, which is the total size of the blocks in the subtree; 2. A pointer to the next block in the tree; B. The block tree structure. Notice that each block represents a part of the current sequence, with the inserted part that comes after it, as in the original list structure. Additionally, the size attribute for the root block would be $k = k' + (y + j) + k''$, which is the size of the left and right subtrees (k' and k'') together with the size of the root node, which is $y + j$.

Finding the first affected block in the block tree

As in the block list algorithm, we use the variable q to find the block in which a given event starts. For generality, we will assume we are currently at block i, even though we start the search at the root block. If the size attribute of the left descendant block is bigger than q, we descend to the left descendant block. Otherwise, and assuming we are at block i, we subtract the size attribute of the left descendant of block i from q and compare it to the total length (T_i) of the block. If $q < T_i$, the event starts at the current block. Otherwise, we subtract the total block size from q and descend to the right descendant block. We continue this process recursively until we reach the affected block. Of note, the "next" attribute allows us to efficiently handle deletions that affect multiple blocks.

Supplemental Information S4: MSA generation using the super-sequence

Generating the MSA from a set of simulated sequences: detailed explanation

The super-sequence is initialized as a linked list of length l, the length of the root sequence (Figure S4A). We then generate the pointer sequence assigned to the root node. Unlike the integer indexing shown in Figure 3, our implementation uses direct pointers from each position in the pointer-sequence to corresponding entries in the super-sequence. This direct pointer approach clarifies the name "pointer sequence."

Branch processing. We traverse the phylogenetic tree (pre-order). Given an insertion event, the pointers of the parent pointer sequence allow us to update the super-sequence in the right location relative to all other positions within the super-sequence. We also update the current pointer sequence with the insertion event. In case of a deletion event, we only update the current pointer sequence, i.e., we remove the pointers for positions that were deleted (Figure S4A).

Final indexing: Next, we assign an index to each node in the super-sequence (numbering the columns of the final MSA), excluding gap-only columns that arise when deletion events independently remove sites from all leaf sequences (Figure S4B).

Alignment determination: For each pointer-sequence, the indices of its referenced nodes reveal gap positions, consecutive indices indicate no gaps, while non-consecutive indices (i.e., a jump > 1) signify gaps (Figure S4C).

This approach ensures that the final alignment accurately reflects the sequence history. Note that this method requires copying the pointer sequence on each indel event. This copying can be averted with the bookkeeping approach, by iterating through the complete block list once to create the child pointer sequence, going through the predecessor pointer sequence and copying only the necessary references to the super-sequence while adding the new insertions to the super-sequence.

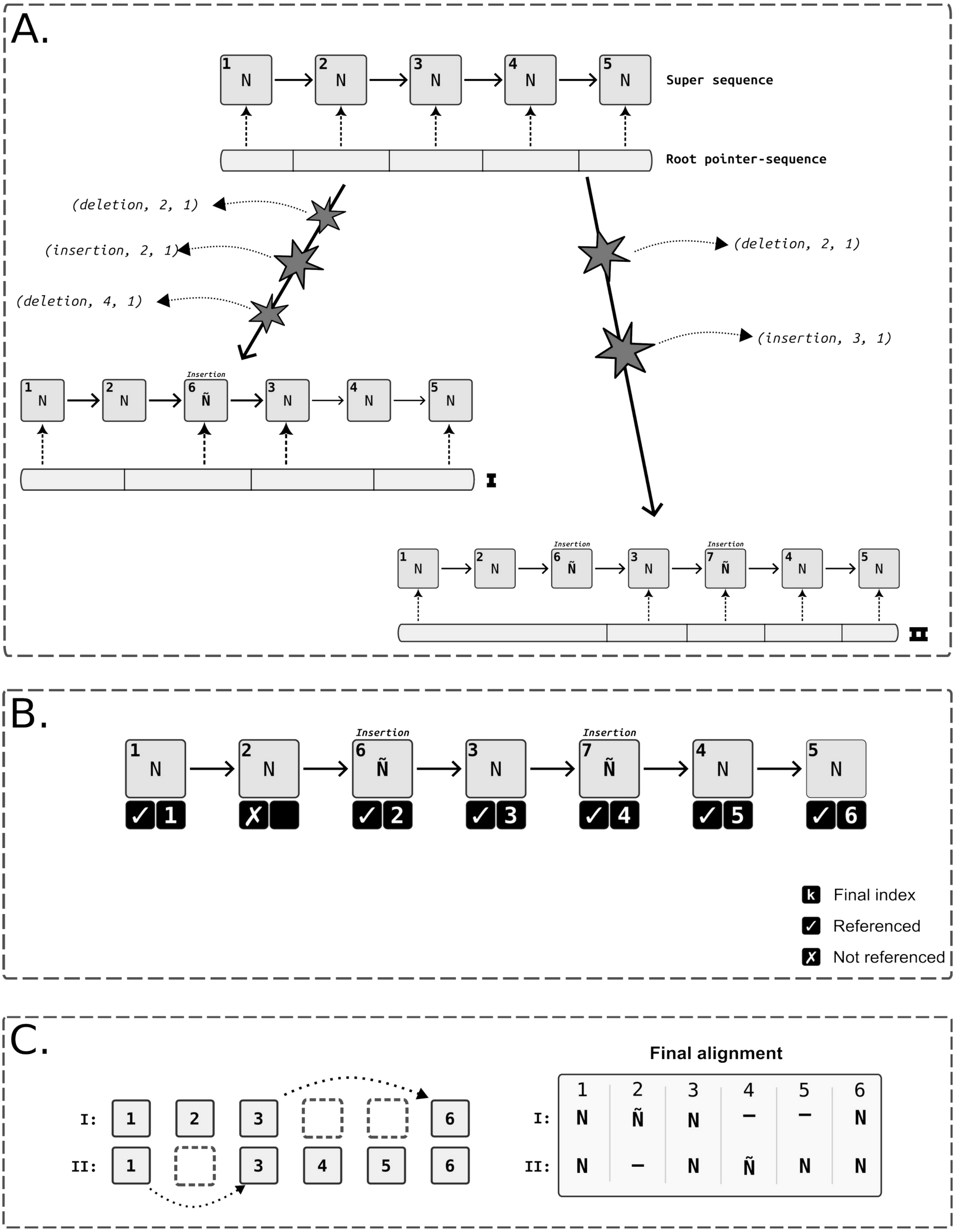

Figure S4. MSA generation using a super-sequence. A. The root pointer-sequence is a list of references to the super-sequence nodes, in this case, we start with a sequence of five characters; We assume that the simulation occurred along a tree with two leaves I and II. In the lineage leading to leaf I, two deletions have occurred: the 2nd and 4th positions. The corresponding nodes in the pointer sequence are deleted. Assume also that after this deletion, an insertion occurred between nodes 2 and 3. We thus update both the super-sequence and the pointer sequence. In the lineage leading to leaf II, a deletion even occurred, removing node 2 of the pointer sequence. Next, an insertion occurred between positions 3 and 4. The final super-sequence is found at leaf II containing all insertion events that occurred along the tree. B. The final super-sequence containing all inserted nodes. On the lower left of each node, we can see if this node was referenced by any of the leaf "pointer-sequences". On the lower right of the node, the absolute index of the node when counting from the first referenced node in the super-sequence; C. The computation of the final MSA using the leaf pointer-sequences.

Table S1.

The mean runtime ± standard deviation in milliseconds across the different simulation methods and root length scenarios. Each column represents a different root length, and each row a different simulation method.

	50	100	500	1,000
Naïve	1.30±0.21	3.87±0.65	74.90±4.60	306.57±19.60
Block list	1.29±0.19	2.41±0.50	12.16±0.72	25.94±2.15
Block tree	1.51±0.26	2.73±0.54	13.68±1.51	29.10±3.06

Supplemental Information S5: Runtime analysis for the SpartaABC inference on empirical data (chiropterans vs. other mammals).

For each of the 47 datasets, we ran the Python versions of the naive and block-list based indel simulators. The ratio between the running times between the two simulators, for the chiropteran data, was on average 9.6. For the other mammals, the corresponding value was 17.5. We used the computed ratios for each dataset to estimate the running time of the SpartABC inference had it been implemented using the naive simulation approach. Of note, the simulator within SpartaABC simulates indels only, and is implemented in the C++ programming language.
